# Vis-NIRS as an auxiliary tool in the classification of bovine carcasses

**DOI:** 10.1371/journal.pone.0317434

**Published:** 2025-01-23

**Authors:** Gabriela Zardo Pereira, Gabriel de Morais Pereira, Rodrigo da Costa Gomes, Gelson Luís Dias Feijó, Lucy Mery Antonia Surita, Marília Williani Filgueira Pereira, Gilberto Romeiro de Oliveira Menezes, Jaqueline Rodrigues Ferreira Cara, Luis Carlos Vinhas Ítavo, Saulo da Luz e Silva, Melissa Amin, Marina de Nadai Bonin Gomes

**Affiliations:** 1 College of Veterinary Medicine and Animal Science, Federal University of Mato Grosso do Sul, Campo Grande, Mato Grosso do Sul, Brazil; 2 Embrapa Beef Cattle, Brazilian Agricultural Research Company, Campo Grande, Mato Grosso do Sul, Brazil; 3 Department of Animal Science, Federal Rural University of Semi-Arid Region, Mossoro, Rio Grande do Norte, Brazil; 4 College of Animal Science and Food Engineering, University of São Paulo, Pirassununga, São Paulo, Brazil; University of Agriculture Faisalabad, PAKISTAN

## Abstract

This work aimed to evaluate the use of Visible and Near-infrared Spectroscopy (Vis-NIRS) as a tool in the classification of bovine carcasses. A total of 133 animals (77 females, 29 males surgically castrated and 27 males immunologically castrated) were used. Vis-NIRS spectra were collected in a chilling room 24 h *postmortem* directly on the hanging carcasses over the *longissimus thoracis* between the surface of the 5th and 6th ribs. The data were evaluated by principal component analysis (PCA) and the partial least squares regression (PLSR) method. For the prediction of sex, the best model was the Standard Normal Variate (SNV) because it presented a relatively high coefficient of determination for prediction, presenting a percentage of correctness of 75.51% and an error of 24.49%. Regarding age, none of the models were able to differentiate the samples through Vis-NIRS. The findings confirm that Vis-NIRS prediction models are a valuable tool for differentiating carcasses based on sex. To further enhance the precision of these predictions, we recommend using Vis-NIRS equipment with the full infrared wavelength range to collect and predict sex and age in intact beef samples.

## Introduction

The Brazilian beef market has undergone a series of transformations in the last decade, many of which are due to challenges that have placed Brazil as a major contributor in the global production and supply of beef.

In this scenario, the Brazilian beef processing industries started to adopt systems of classification and typification of carcasses capable of distinguishing products with different characteristics and added value in their slaughtering routines, putting pressure on producers to organize and invest in the production of better-quality animals. An obstacle in this traditional system of classification of carcasses is that after slaughter, with the removal of non-carcass components such as the head and reproductive organs, it becomes more difficult to identify sexual characteristics and the age of the animals. In meat, after deboning, this information would be almost impossible to determine and could only be identified with more sophisticated tests, such as DNA tests, which is impractical in the routine of a slaughterhouse industry [1].

Techniques that assist the collection and control of information within the industry must be tested to meet the most varied requirements for quality of the consumer market. Technologies such as visible and near-infrared spectroscopy (Vis-NIRS), which is a nondestructive, economical, simple, rapid, and safe method for evaluating meat quality attributes and chemical composition, have been used as alternatives to traditional quality assessment methods [2–4], with a percentage of correct answers above 80% in most cases [5–7].

Spectroscopy is an analytical method used to identify physical properties and determine chemical composition based on a substance’s unique spectrum [8]. The technique is based on the premise that different chemical bonds in organic matter, when irradiated, absorb and emit light at different wavelengths [9] and in conjunction with multivariate data analysis, can provide information on several chemical and physical parameters of meat simultaneously [10, 11].

Vis-NIRS equipment for meat analysis includes the visible range (Vis—400 to 800 nm) and the near infrared (NIR—near infrared, 800–2500 nm) [12]. In the infrared region, radicals such as -OH, -NH and -CH vibrate intensely [8] and recording the electromagnetic radiation absorbed from these molecular bonds produces unique spectra, which include data related to the chemical and physical properties of organic molecules, i.e. important information about their composition [11, 13]. As these groups are typical food components (water, protein, lipid and carbohydrate) the Vis-NIRS method can be used for a range of qualitative meat analyses, from predicting chemical composition, fatty acid composition, as well as industrially important characteristics such as pH, color, water retention capacity, tenderness and sensory attributes [3, 5–7, 11, 14, 15].

Vis-NIRS technology has been widely used to predict the qualitative characteristics of meat from production animals. In a study to predict and classify beef tenderness based on shear force (SF), [3] predicted SF values with an R^2^p of 0.46 and classified 100% of hard samples (>45N) and incorrectly classified all soft samples (≤45N). [4] used spectroscopy to predict to SF values and categorize pork by tenderness (soft or hard) and predicted SF values with an R^2^p of 0.25. In turn, [16] evaluated bovine neck skin samples to predict age using a PLSR model developed with the lowest root mean square error of prediction (RMSEP) of 2.0 years and R^2^ of 0.63.

The objective of this study was to test the Vis-NIRS accuracy to classify carcasses based on sex, age, and castration type.

## Materials and methods

The study was carried out at the Meat Processing and Technology Laboratory “QUALICARNES” of the Federal University of Mato Grosso do Sul (UFMS) and in slaughterhouses located in Campo Grande, Terenos and Rochedo, all located in the state of Mato Grosso do Sul, Brazil.

### Animals and meat samples

A total of 133 animals (77 females, 29 surgically castrated males and 27 immunologically castrated males (Bopriva®), comprising progenies from Nelore, ½ Valdostana x ½ Nelore, ½ Angus x ½ Nelore, ½ Caracu x ½ Nellore dams mated with Nelore, Caracu, Pardo Suíço, and Brahman, were subjected to carcass analysis.

The animals were from herds of UFMS and Embrapa Beef Cattle, both located in Campo Grande, MS, Brazil. The animals were slaughtered at two commercial abattoirs located at Terenos and Rochedo, MS, Brazil, in accordance with the Humanitarian Slaughter Guidelines (as needed by Brazilian law), and carcass processing followed the common industry practice adopted in Brazil [17].

At the time of slaughter, each carcass was identified individually in accordance with the identification of the live animal and classified according to sex, in accordance with the standards established by the Ordinance No. 612 of October 5th, 1989, of the Ministry of Agriculture, Livestock and Supply of Brazil (MAPA), which establishes the standards for the Classification and Typification of Bovine Carcasses [18].

Sex was verified by observing sexual characteristics, establishing the following categories: females (F), surgically castrated males (C), and immune castrated males (I). Furthermore, information regarding the castration type was obtained and verified with the animal’s history prior to slaughter. The age of the animal was calculated in months by subtracting the age at slaughter from the date of birth. After slaughter, the half carcasses were chilled at 0–2°C for one day (24 h) and then sawed between the 5^th^ and 6^th^ ribs, where the front and rear quarters of the carcass are separated in Brazilian industries.

### Vis-NIRS spectra measurements

Spectral scanning was carried out directly on the chilled left half carcasses over the *longissimus thoracis* (LT) between the surface of the 5th and 6th ribs using a portable visible/near-infrared spectrophotometer device comprising an EPP2000-CXR-Srs model for the 220 to 1110 nm wavelength range and another EPP2000-InGaAs-512 for the 900 to 1700 nm range (Stellarnet Inc, Florida, USA), using SpectraWiz software (StellarNet Inc., Tampa, FL). The reference and dark spectra were stored before the sample spectrum was acquired [4].

The spectra data was collected in transmittance mode in the wavelength range from 400 a 1395 nm at intervals of five consecutive wavelengths, with an average of 20 scans for each spectrum, thus generating 200 data points [3, 19]. Three replicated spectra were obtained from each sample at different points, side by side, spaced 1 cm apart. The scanned area was circular in shape, with a diameter of three centimeters, corresponding to a total/sample of 21.12 cm^2^. The intensity of each sample’s spectrum was determined from the average of the three scans.

### Data analysis

Multivariate analysis of the spectral data was carried out using Unscrambler X v.10.3 software (CAMO Software AS, Oslo, Norway) [3]. For PCA, the raw spectra were used to detect clusters of samples and possible outlier data before using the dataset to develop PLSR models [20, 21]. The clean database was randomly divided by the Unscrambler X software into a calibration set with 80 samples (60% of the database) and a validation set with 53 samples (40% of the database). The PLSR was used to develop the prediction equations for the traits (sex, age and type of castration) using Vis-NIRS spectra. The calibration set was evaluated using internal cross-validation by applying the following metrics: the coefficient of determination for calibration (R^2^) and the standard error in cross-validation (SECV). The prediction equations developed from the calibration set were also evaluated using independent test set samples (test set validation). The validation errors from the test set were combined in the coefficient of determination for prediction (R^2^) and the standard error in prediction (SEP) [22]. The performance and reliability of the prediction equations were also evaluated using the residual prediction deviation (RPD) value, as proposed by [4, 23, 24].

Spectral preprocessing algorithms were applied (no pre-processing (none), Baseline Correction (Baseline), SNV, Multiplicative Scatter Correction (MSC), first derivative (1st Der), and second derivative (2nd Der) to reduce physical variability corresponding to light scattering and path length variation to highlight chemical differences between the samples and distinguish the features of interest throughout the spectral range [25]. After the development of the equations, the one with the highest R^2^ in the calibration was tested for its predictive accuracy (R^2^ prediction). The real and predicted values were used to classify the carcasses for age, sex and type of castration, thus obtaining the percentage of correct classification of the carcasses from the Vis-NIRS spectra compared to the reference values obtained in the evaluation of the carcasses at slaughter.

## Results and discussion

The descriptive statistics are shown in Table 1, with the number of animals used (n), and the respective ages in months, as well as the mean, maximum and minimum values, and standard deviations (SD) for each sex used (females, surgically castrated males and immune castrated males).

**Table 1:** Descriptive statistics of the number of animals (n) and the respective ages in months of cattle used in Vis-NIRS datasets to predict the sex, age and types of castration.

		Age (months)			
Sex	n	Minimum	Maximum	Mean	SD
**Females**	77	21.93	25.70	24.43	0.62
**Surgically castrated males**	29	23.57	26.40	24.83	0.72
**Males**	27	23.00	26.13	25.03	0.71

SD: standard deviation.

In this study, no model was able to accurately predict the age difference between animals (Table 2). This methodology is still new to meat authentication, but it is already being used in some studies in this field [26–28], there is no existing literature related to cattle on its use in authentication or sex prediction. Only one study has been conducted in relation to the age of the animals [16, 29].

**Table 2:** Variables of partial least squares (PLSR) models and spectra pre-treatment used for classifying carcasses by age using Vis-NIRS.

Age (21 to 26 months)						
Spectral Pre-treatment*	MSEC	R^2^C	CVMSEC	R^2^CV	MSEP	R^2^P
**None**	0.65	0.26	0.77	0.05		
**Baseline**	0.63	0.31	0.75	0.06		
**SNV**	0.66	0.24	0.71	0.16		
**MSC**	0.66	0.24	0.72	0.13		
**1st Der**	0.60	0.36	0.69	0.19	0.90	NA
**2nd Der**	0.64	0.27	0.73	0.10		

*Spectral pretreatment: Spectral preprocessing methods to reduce the influence of sample presentation; MSEC: mean square error of the calibration; R^2^C: calibration determination coefficient; CVMSEC: cross-validation mean square error; R^2^CV: coefficient of determination of cross-validation; MSEP: mean square error of the prediction; R^2^P: prediction determination coefficient; None: No pretreatment; Baseline: Baseline correction; SNV: Standard normal variate; MSC: Multiple scatter correction; 1st Der: First derivative; 2nd Der: Second derivative. NA: not available.

A study conducted by [29] employed near-infrared spectroscopy to categorize animals into two distinct groups: young steers (less than 14 months of age) and castrated adult steers (above four years of age). The study demonstrated a 100% discrimination rate for the animals used in the test. The success found by the authors is due to the differences in intramuscular fat and the water content in the meat of the two groups studied, as it is known that intramuscular fat varies according to the age and castration of the animal [30, 31] and that the percentage of intramuscular fat has a negative correlation with the water content of meat samples [32, 33]. Therefore, these chemical differences in meat between the two groups were responsible for the different absorption peaks, thus being able to differentiate samples from young or adult cattle.

In the present study, the medium values of intramuscular fat (data not presented) were 3.97 ± 1.18, 3.59 ± 1.42, and 3.21 ± 1.41 for females, surgical and immune castrated males, respectively. However, we cannot use this information to justify our results as made by [29] because the wavelength (1100 to 2500 nm) and type of sample (minced meat) used by the authors differs from ours (400 to 1395 nm, intact samples, respectively). It is widely acknowledged that the chemical properties of meat can be accurately predicted by spectra collected in the infrared region, mainly between the wavelengths 1100 and 1700 nm [34] in minced meat samples. This because the structure of the muscle fibers in intact samples could potentially cause a diffraction phenomenon [20, 34], which might then affect the way that spectra are absorbed and reflected by the meat and consequently influencing in the accuracy of predictions.

The portable Vis-NIRS equipment, as used in the experiment, has the advantage of being suitable for use in routine industry settings with relatively low cost [35], which is an attractive feature compared to static infrared equipment. Furthermore, we investigated the possibility of predicting the age and sex/gender of animals in intact samples, streamlining the process and integrating this capability into abattoir routines, should it be feasible.

**Fig 1:**
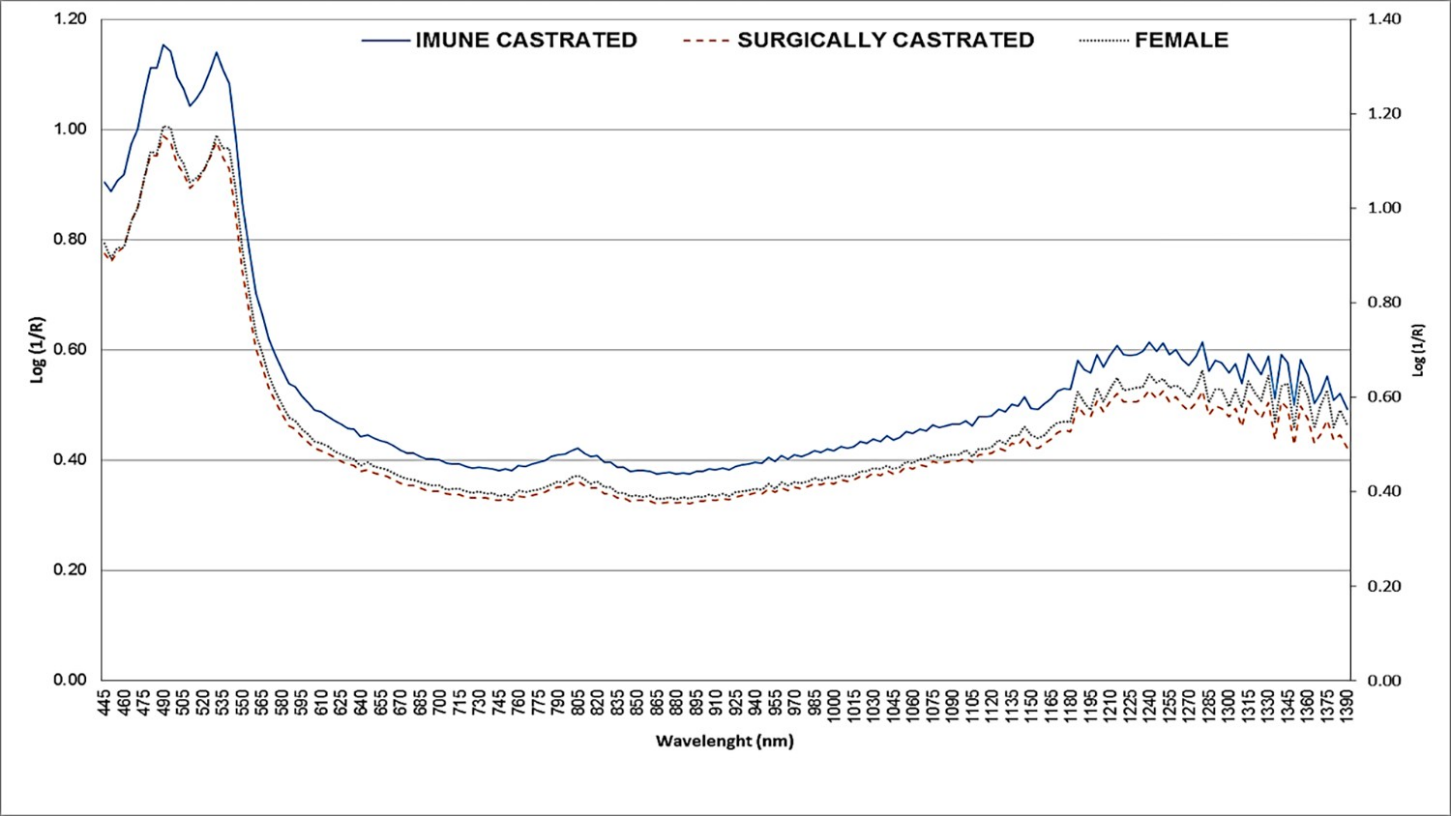
Average spectra [log (1/R)] corresponding to the meat samples obtained from female (n = 77), surgically castrated (n = 29) and immune castrated (n = 27).

The best model for differentiating sex through Vis-NIRS was the SNV, with less probability of error and greater accuracy when compared with the other tested models (Table 3 and Fig 1). The SNV is a normalization method that transforms data with a mean of 0 and a standard deviation of 1. This transformation is applied to each spectrum individually and serves to enhance the important signals while minimizing the impact of unwanted artifacts, thereby enhancing the probability of association of the spectra with the reference variable. Although SNV pretreatment yielded higher R^2^ values for calibration, cross-validation, and predictions, in our study, these values were only marginally superior to those generated by the untreated spectra. This indicates that the spectral data were of high quality and sufficiently robust to differentiate bovine carcasses by sex.

**Table 3:** Variables of partial least squares (PLSR) models and spectra pre-treatment used for classifying carcasses by sex and type of castration using Vis-NIRS.

Sex (females, surgically castrated males and imune castrated males)						
Spectral Pre-treatment*	MSEC	R^2^C	CVMSEC	R^2^CV	MSEP	R^2^P
**None**	0.39	0.77	0.50	0.63	0.62	0.41
**Baseline**	0.41	0.74	0.53	0.60		
**SNV**	0.35	0.81	0.48	0.64	0.68	0.27
**MSC**	0.36	0.80	0.48	0.65		
**1st Der**	0.43	0.71	0.47	0.66		
**2nd Der**	0.36	0.50	0.79	0.63		

* Spectral pretreatment: Spectral preprocessing methods to reduce the influence of sample presentation; MSEC: mean square error of the calibration; R^2^C: calibration determination coefficient; CVMSEC: cross-validation mean square error; R^2^CV: coefficient of determination of cross-validation; MSEP: mean square error of the prediction; R^2^P: prediction determination coefficient; None: No pretreatment; Baseline: Baseline correction; SNV: Standard normal variate; MSC: Multiple scatter correction; 1st Der: First derivative; 2nd Der: Second derivative.

Our findings differ from those of a previous study on chickens [27], which found no difference in any model for predicting sex through Vis-NIRS. It is possible that the discrepancy is due to the younger age of the chickens in that study (maximum 16 weeks) and their lack of reaching the maturity period (above 18 weeks). The most significant change in body composition during animal growth occurs after puberty and may be related to the proportion of water and fat content in the empty body. It seems that after puberty, there may be a notable increase in fat accretion and a corresponding decrease in muscular growth rate, being the fat deposition an indicator of puberty and the start of the adult phase [36]. Accordingly, the study by [27] indicates that the body composition of males and females was likely comparable, resulting in minimal discernible differences in light absorption that could be utilized to differentiate male and female meats using NIRS spectra.

**Fig 2:**
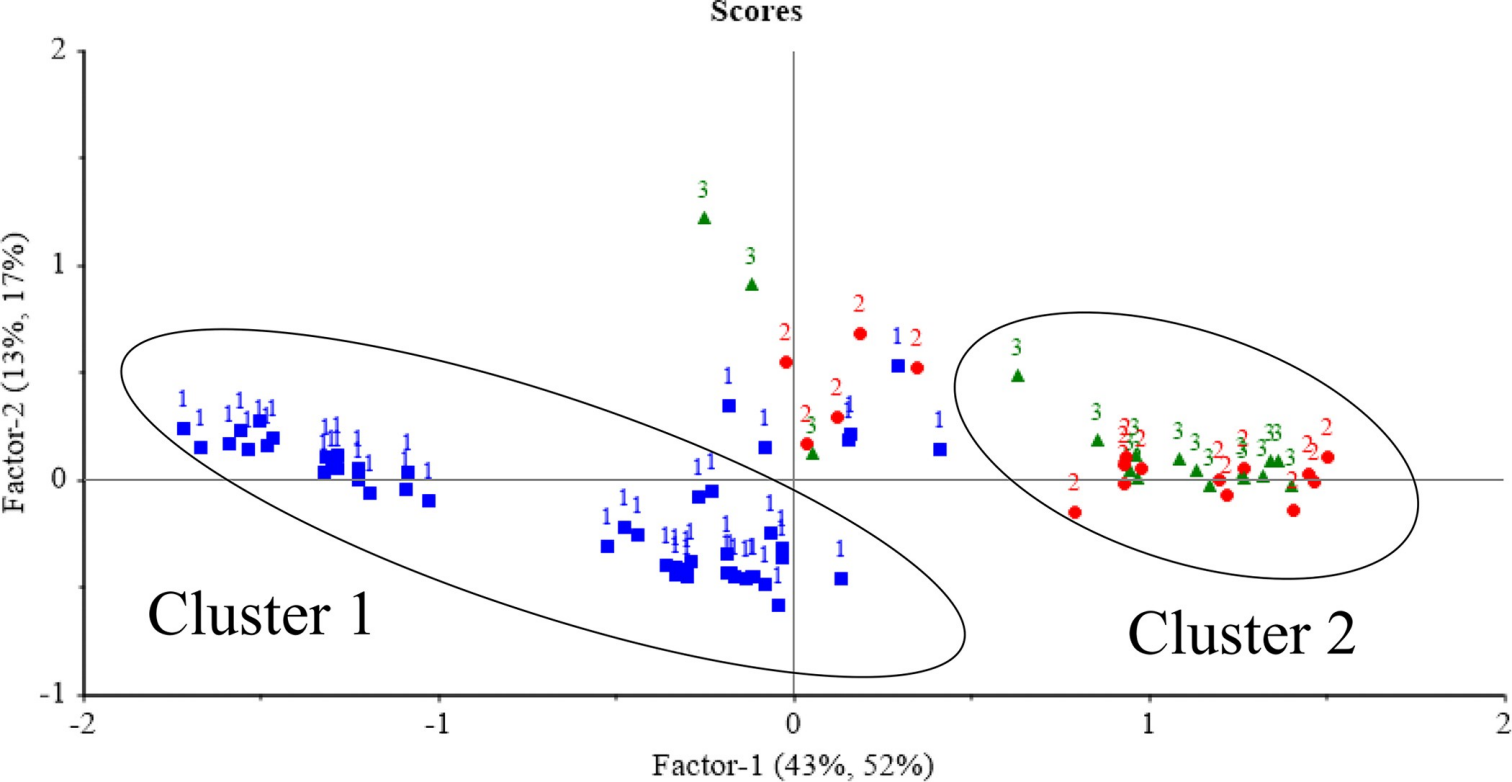
Score plot of factors 1 and 2 of partial least square regression models (PLSR) used to identify samples from females (1), surgically castrated males (2) and immune castrated males (3), using Vis-NIRS spectra.

As shown in Fig 1 the average spectra of immune and surgically castrated males are very similar, indicating that, in the wavelength evaluated, no differences between two classes were possible to be identified. For females, the medium spectra presented higher absorbance values than castrated males. Distinct pathways of light absorption by samples resulted in two different clusters (1 for females and 2 for males samples) in the PLSR score plot (Fig 2), indicating that there is no difference in the meat composition of castrated groups, as they have very similar, being grouped in the same cluster.

It is also worth noting that, as illustrated in Fig 2, some samples appear to deviate from the two clusters, suggesting a potential for further investigation. It is possible that this may occur in samples with a chemical composition that differs from the established group and is not aligned with the factors of the model. The difference between the absorption peaks of the male and female samples can be explained by the chemical composition of the meat due to differences in the meat composition of male and female bovines at the same age [34] since females have a greater deposition of subcutaneous and intramuscular fat than males [35]. These factors influence the water content of the samples [32] and absorption through spectrophotometry, making it possible to distinguish meat from animals of different sexes by Vis-NIRS.

**Fig 3:**
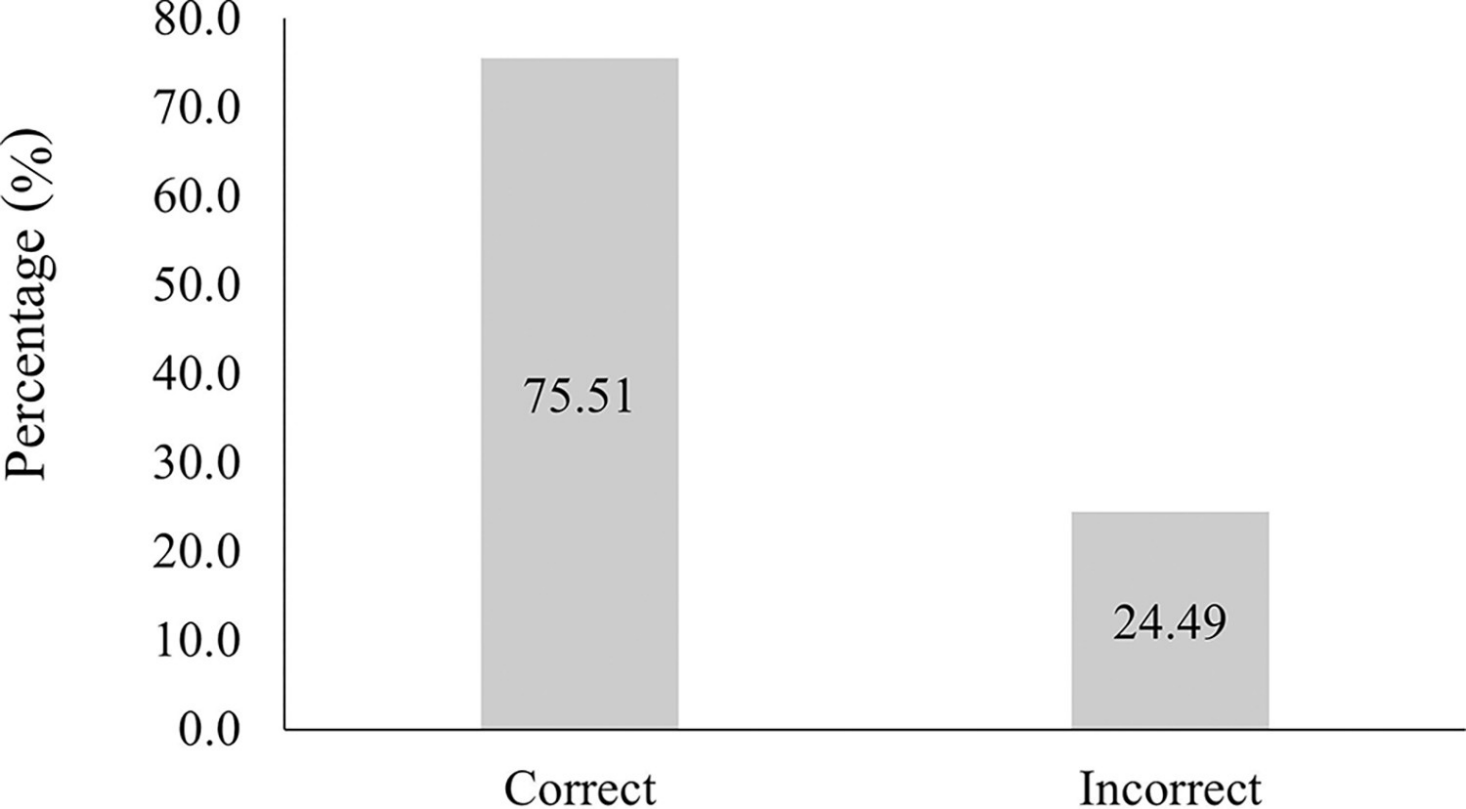
Percentage of successes and errors in predicting sex (male and female) through Vis-NIRS using the SNV preprocessing model.

On the other hand, as previously discussed, the chemical composition of the meat can be more accurately predicted in wavelengths in the infrared region and in minced samples [34]. However, it should be noted that the equipment used in this study comprises just a part of this spectrum (800 to 1395 nm), and that this was insufficient to distinguish the samples accurately, resulting in an error of classification of the test samples of 24.49% (Fig 3).

The use of spectroscopy to determine the chemical composition of meat in the near infrared region has already been established with skill proven by numerous studies that found high determination coefficients (R^2^), between 0.87 and 0.99 [15, 37, 38].

In this way, we recommend that furthers studies using portable Vis-NIRS equipment with wavelengths comprising all visible and infrared region (400 to 2500 nm) should be conducted to elucidate the improvement of correct classifications of sex and age in intact meat samples, augmenting the feasibility of adoption of this technology by the industries.

## Conclusion

The spectra collected in the visible and near-infrared regions were unable to accurately classify intact meat samples in terms of age. Furthermore, they were unable to differentiate between surgically castrated and immune males and females. As a result, only 75.51% of the samples were correctly classified in this regard. It is recommended that further studies be conducted to encompass all regions of the visible and infrared spectra in order to elucidate the potential for enhanced correct classifications by sex and age in intact meat samples.

## Supporting information

S1 Fig Mean and standard deviation. (TIF)

S2 Fig Standard deviation of tissue. (TIF)

S3 Fig Database. (XLSX)

S4 Fig Database. (XLSX)
