# Supplementary figures and images for: Vis-NIRS as an auxiliary tool in the classification of bovine carcasses
Source: PLoS One. 2025 Jan 23;20(1):e0317434. doi: 10.1371/journal.pone.0317434 (PMC11756776; doi:10.1371/journal.pone.0317434)

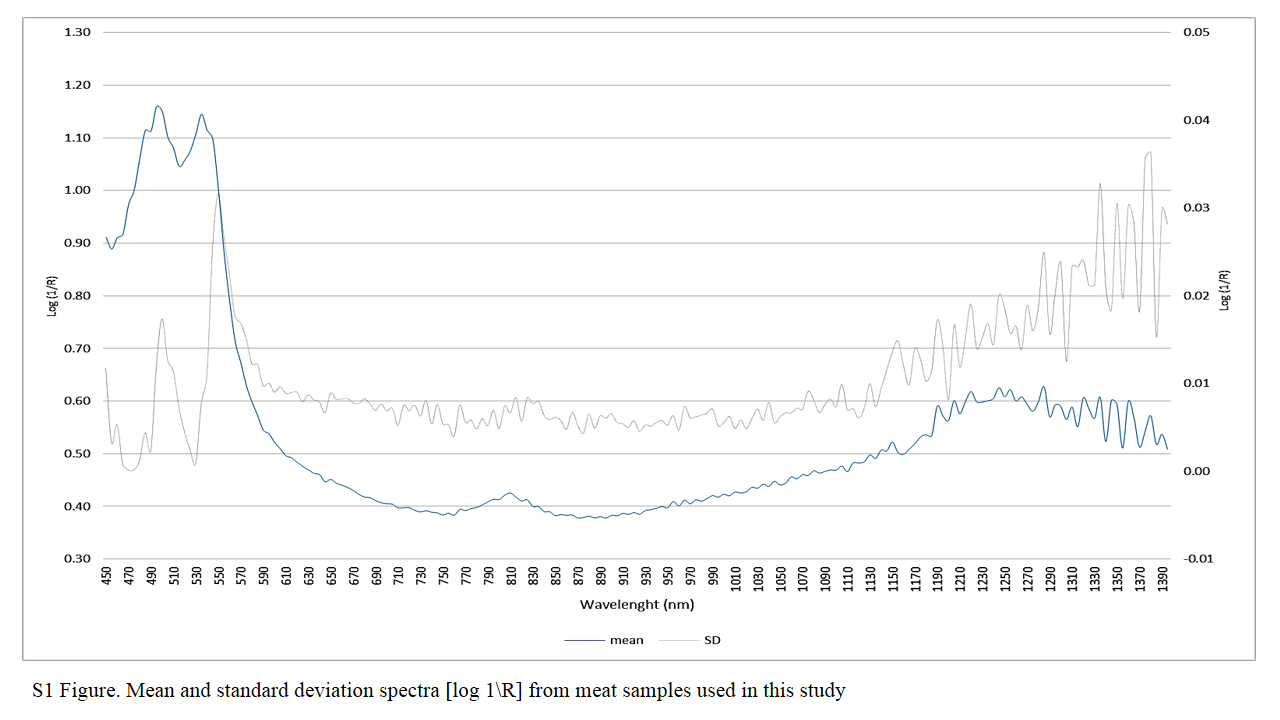

Supplement: S1 Fig — (TIF) [file pone.0317434.s001.tif]

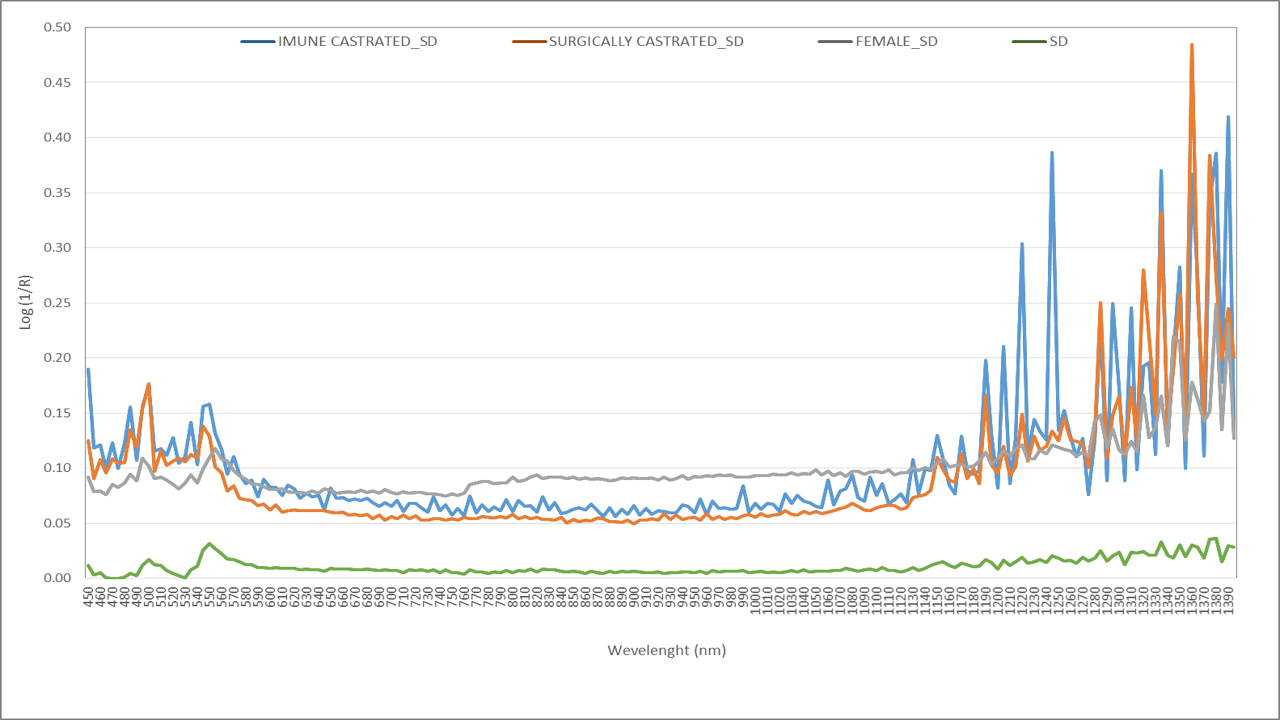

Supplement: S2 Fig — (TIF) [file pone.0317434.s002.tif]
